# Evaluating adverse effects of environmental agents in food: a brief critique of the US FDA’s criteria

**DOI:** 10.1186/s12940-023-00971-2

**Published:** 2023-04-21

**Authors:** Laura N. Vandenberg, R. Thomas Zoeller, Gail S. Prins, Leonardo Trasande

**Affiliations:** 1 Department of Environmental Health Sciences, School of Public Health and Health Sciences, University of Massachusetts – Amherst, 240G Goessmann, 686 N. Pleasant Street, Amherst, MA 01003 USA; 2 Department of Biology, University of Massachusetts Amherst, Amherst, MA USA; 3 Visiting Professor, Örebro University, Örebro, Sweden; 4 Department of Urology, School of Medicine, Division of Epidemiology & Biostatistics, School of Public Health, University of Illinois at Chicago, Chicago, USA; 5 Departments of Pediatrics, Environmental Medicine, and Population Health, New York University School of Medicine, New York, NY USA

**Keywords:** Hazard assessment, Pharmaceutical, Lupron, Gender difference, Transparency, Endocrine disrupting chemical

## Abstract

**Background:**

In the US, the Food and Drug Administration (US FDA) is charged with protecting the safety of food from both pathogens and chemicals used in food production and food packaging. To protect the public in a transparent manner, the FDA needs to have an operational definition of what it considers to be an “adverse effect” so that it can take action against harmful agents. The FDA has recently published two statements where, for the first time, it defines the characteristics of an adverse effect that it uses to interpret toxicity studies.

**Objective:**

In this brief review, we examine two recent actions by the FDA, a proposed rule regarding a color additive used in vegetarian burgers and a decision not to recall fish with high levels of scombrotoxin. We evaluated the FDA’s description of the criteria used to determine which outcomes should be considered adverse.

**Overview:**

We describe three reasons why the FDA’s criteria for “adverse effects” is not public health protective. These include an unscientific requirement for a monotonic dose response, which conflates hazard assessment and dose response assessment while also ignoring evidence for non-linear and non-monotonic effects for many environmental agents; a requirement that the effect be observed in both sexes, which fails to acknowledge the many sex- and gender-specific effects on physiology, disease incidence and severity, and anatomy; and a requirement that the effects are irreversible, which does not acknowledge the role of exposure timing or appreciate transgenerational effects that have been demonstrated for environmental chemicals.

**Conclusions:**

The FDA’s criteria for identifying adverse effects are inadequate because they are not science-based. Addressing this is important, because the acknowledgement of adverse effects is central to regulatory decisions and the protection of public health.

The US Food and Drug Administration (FDA) is charged with protecting the safety of the nation's foods [1] from pathogens, such as *E. coli*, as well as from chemicals used in the production of food and food packaging. Both chemicals and pathogens can cause adverse health effects, but the specific adverse effects of their exposures are very different. For example, the US Centers for Disease Control and Prevention (CDC) estimates that 48 million Americans will become ill each year from pathogens and “unspecified agents” causing acute foodborne illnesses [2]; of these, 3,000 people (about 0.1% of the American population) will die. In contrast, greater than 95% of Americans (hundreds of millions of people) are exposed every day to chemicals found in food, and exposures to these chemicals have been shown to increase the risk of chronic, rather than acute, health effects [3].

An agent has the potential to produce an adverse effect by virtue of its fundamental properties (e.g., it may be a carcinogen, a developmental toxicant, etc.). Thus, a central feature of “risk assessment” is the identification of an “adverse effect” caused by the hazard, and the “potency” of the hazard on that adverse effect. The adverse effect is a health “endpoint”, or measurement of something that a regulatory agency would consider to be important enough to regulate a chemical or pathogen to protect human health. For some pathogens, like specific strains of *E. coli* that cause death, the adverse effects of exposure are relatively straightforward. For other hazards, like chemicals such as phthalates or perfluoroalkyl and polyfluoroalkyl substances (PFAS) that are commonly found in food and food packaging [4, 5], exposures have been associated with increased incidence of various chronic diseases (or biomarkers of disease) [6–8]; in these cases, determining which measurable endpoints affected by environmental exposures are “adverse” is less simple [9, 10] but remains critical.

Until recently, the FDA has not provided an objective, transparent explanation of what the agency considers an “adverse effect”[11]. This is important because an observed effect must be considered adverse for it to be used in a regulatory decision; in other words, if there is no adverse effect, no risk is anticipated and no action will be taken by a regulatory agency. The opacity of what the FDA considers an adverse effect makes it difficult for the consuming public to know whether any risk assessment is sufficient to protect public health. In addition, identification of the most sensitive adverse effect is critical for public health protection [10]. For example, if an agent causes death at one dose, but contributes to the incidence of type 2 diabetes at a million-fold lower dose, it is not public health protective to regulate on the basis of the exposure that causes death.

The FDA has recently written two decisions in which it defined the general characteristics it considers as an “adverse effect”. These declarations raise serious concern about how the agency defines and identifies adverse outcomes so that it can set total daily exposure limits that are health protective. In the first, the FDA was responding to objections raised by the Center for Food Safety on the agency’s proposed final rule regarding a color additive used in vegetarian burgers to make them appear more like ground beef [12]. The FDA wrote: “For an observed effect to be toxicologically relevant (i.e., potentially adverse), a clear dose–response should be seen (e.g., increasing the dose of a test substance causes an increase in the observed effect in the test subjects), and the observed effect should occur in both sexes of test species” [12] [emphasis added]. In the second example, the FDA investigated poisoning by scombrotoxin, which occurs when fish are not properly refrigerated and high levels of histamine are produced [13]. The FDA declined to pursue a mandatory recall of affected fish because “scombrotoxin fish poisoning causes temporary or medically reversible adverse health consequences” such as nausea, diarrhea, blurred vision, and respiratory distress [emphasis added] [14]. The public would likely question whether these outcomes would be considered inconsequential, and there is no further justification for why a medically treatable outcome should be ignored by a regulatory agency charged with protecting the public’s health. Furthermore, in some cases, individuals exposed to scrombotoxin can experience life-threatening anaphylactic reactions or cardiovascular conditions requiring hospitalization, especially if these individuals have other conditions that increase their medical vulnerability [15].

These actions by the FDA provide an explicit description of the criteria used by the agency to determine which outcomes, induced by chemicals, agents, or pathogens in food, are considered adverse. They shed light on a process that has been criticized for its lack of transparency both by the regulated and scientific communities [16]. But with this transparency comes a stunning realization that FDA is not basing its risk assessments on a logical footing. Rather, there are several well-established scientific observations that require rejection of these criteria because they are neither health protective nor adequate. These observations are:

1. Requiring a “clear dose response” where “increasing the dose of a test substance causes an increase in the observed effect” (e.g., monotonicity). This is problematic for two reasons. The first is pragmatic: it conflates the dose response evaluation with hazard identification itself, which are two separate and independent steps in a risk assessment. In other words, with the FDA’s current approach, a chemical or agent will not be identified as a hazard unless it is considered a potential risk. The second problem is scientific: in defining a “clear dose response” as a monotonic relationship between exposure and effect, the FDA ignores well-known non-monotonic relationships that can exist between dose and effect for a range of substances including environmental chemicals, pharmaceuticals, hormones, vitamins, and essential nutrients [17, 18]. The FDA’s own data on pharmaceuticals recognizes that low doses can induce undesirable effects that are opposite to those observed at higher doses. An example comes from Lupron, a drug that acts as an agonist for the Gonadotropin Releasing Hormone (GnRH) receptor which is used for the treatment of numerous hormone-mediated diseases including prostate cancer, endometriosis, female infertility, polycystic ovarian syndrome, and uterine fibroids. When a patient with a disease like endometriosis is first administered Lupron, the lower circulating concentrations can increase the adverse symptoms associated with endometriosis including ectopic growth of uterine tissue [19], whereas continued exposure producing higher circulating levels of Lupron is used to manage the disease [20]. Similarly, when breast cancer patients first start taking the drug tamoxifen, low serum concentrations can increase bone and tumor pain as well as localized tumor flare (i.e., growth) whereas at higher serum concentrations, tumor growth is inhibited [21, 22].
  A related issue is that the FDA has dismissed the presence of non-monotonic dose responses for chemicals found in food. For example, after stakeholders claimed that “FDA does not recognize that some substances have a greater adverse effect at low doses than at medium doses, which is one example of what is referred to as a nonmonotonic dose–response relationship”, the FDA responded in a November 2022 report from the US Government Accountability Office [23] that “they have reviewed the scientific literature but found that the available studies do not support concerns about health effects associated with nonmonotonic dose–response relationships.”
2. Requiring that the effect is observed in both sexes. This is problematic because it does not acknowledge that males and females are physiologically and anatomically different, and therefore provides no guidance for how the FDA might consider adverse effects that occur in the gonads or primary/secondary sex organs and other tissues that exhibit sex-specific responses. It fails to recognize that disease incidence, course of progression, and severity differ between males and females for a number of conditions including cardiovascular diseases, autoimmune diseases, neurological and psychiatric disorders, asthma, and some cancers [24, 25]. Males and females also experience pain differently [26], have differences in the sizes of different brain regions as well as the age at which brain volume reaches its peak [27], and differences in their gut microbiota, with implications for disease susceptibility [28]. Finally, although females have long been excluded from many clinical trials, there is strong evidence for sex differences in the metabolism of hormones, drugs, and other chemicals [29, 30].
3. Requiring that adverse effects be irreversible. This is problematic for two reasons. First: it assumes that exposures are transient. Yet, if the inducing agent is found in foods consumed daily, effects that would not seem severe if they were only induced once (like a headache or diarrhea), could occur more often. This is certainly the case for many chemicals used in food production and packaging (e.g., bisphenols, phthalates, perchlorate) which are found in the vast majority of human urine samples despite relatively short residence time in humans, documenting chronic exposures [31]. Second: the FDA’s reasoning assumed that all individuals are equally vulnerable (e.g., that they would only fall victim to the less serious outcomes like nausea, diarrhea, and blurred vision). With scombrotoxin fish poisoning, it is clear that some individuals are more vulnerable to its effects; in fact, while the dosage of scombrotoxin considered toxic is 1 mg/g fish consumed, many individuals will not have disease responses after exposure to these high doses, whereas other individuals will have reactions after exposures as low as 0.05 mg/g fish [32]. The presumption that all individuals are equally vulnerable is never evidence-based or justifiable. With hormones, hormonally active agents, and teratogens, the effects of even low doses can have permanent effects on embryos, fetuses and/or neonates when exposures occur during vulnerable periods of development, whereas exposures in adults produce effects that are often reversible once exposures cease [33–35]. FDA scientists acknowledged in a peer-reviewed journal article that they understand this basic fact [36]. This very brief analysis of FDA’s recent operational definition of adverse effects demonstrates that these are not scientifically defensible criteria to identify a hazard or characterize the risk posed by these agents. This analysis also indicates that a clear operational definition of an adverse effect is needed to support health-protective actions by the agency.

How are adverse effects defined by other regulatory agencies? The US EPA characterizes an adverse effect as any “biochemical change, functional impairment, or pathologic lesion that affects the performance of the whole organism, or reduces an organism’s ability to respond to an additional environmental challenge” [37]. Other international regulatory agencies use the definition put forth by the Organization for Economic Co-operation and Development (OECD): “a change in morphology, physiology, growth, development or lifespan of an organism which results in impairment of functional capacity or impairment of capacity to compensate for additional stress or increase in susceptibility to the harmful effects of other environmental influences” [38]. Thus, defining an “adverse effect” has not prevented other agencies from identifying hazards, or understanding never-before-seen health effects as they arise, and FDA should be similarly transparent and science-based.

There are many steps that the FDA, and other regulatory agencies, must take to improve chemical safety assessments and risk assessments to protect the public from hazardous agents in food. These include identifying the most sensitive, disease-relevant outcomes measurable in experimental studies [39]; changing practices with regard to the reliance on historical controls, which are often used to dismiss effects of environmental chemicals [40]; abolishing the use of “experts” hired by the chemical manufacturer, and thus relying on individuals with financial conflicts of interest to declare food chemicals “generally recognized as safe”, even in the absence of toxicity data [41]; and implementing strategies to consider the cumulative effects of chemicals, i.e., where multiple chemicals affect common outcomes or the same chemical is present in many products [42]. There is also a need for the FDA to be transparent in the agency’s decision-making processes, publish guidelines for how the agency will assess data and deal with data gaps, and develop improved techniques to systematically review the available literature and use all data in decision-making processes.

It is also clear that the FDA uses very different approaches to evaluate safety of chemicals depending on how they are intended to be used: non-monotonicity is understood in the context of drugs (e.g., Lupron and tamoxifen), but dismissed in the context of food chemicals; outcomes that would be considered adverse “side effects” requiring disclosure to patient administered drugs (e.g., nausea, diarrhea, blurred vision, and respiratory distress) are not automatically considered adverse if induced by food chemicals. This disconnect between the two sides of this federal agency argues against their claim of decision-making that is objective and based on science.

These recent decisions from the FDA have demonstrated that the agency must be compelled to describe the criteria that are used to determine if an outcome is adverse, and those criteria must be scientifically justified. These criteria should also be subject to dynamic revisions as advances in scientific knowledge become available. Protecting the nation’s food supply is a big and important task; this task should be done consistently and correctly. To do so begins with a rational, transparent, consistent, and science-based definition of which health outcomes the FDA deems concerning enough to protect the public.

## Data Availability

Not applicable.
